# Attention Cueing and Activity Equally Reduce False Alarm Rate in Visual-Auditory Associative Learning through Improving Memory

**DOI:** 10.1371/journal.pone.0157680

**Published:** 2016-06-17

**Authors:** Mohammad-Ali Nikouei Mahani, Hojjat Allah Haghgoo, Solmaz Azizi, Majid Nili Ahmadabadi

**Affiliations:** 1 Cognitive Robotics Lab, School of Electrical and Computer Engineering, University of Tehran, Tehran, Iran; 2 School of Cognitive Science, Institute for Research in Fundamental Sciences, Tehran, Iran; 3 Cognition and Perception, Department of Psychology, University of Tübingen, Tübingen, Germany; 4 Occupational Therapy Dept., University of Social Welfare and Rehabilitation Sciences, Tehran, Iran; Centre de Neuroscience Cognitive, FRANCE

## Abstract

In our daily life, we continually exploit already learned multisensory associations and form new ones when facing novel situations. Improving our associative learning results in higher cognitive capabilities. We experimentally and computationally studied the learning performance of healthy subjects in a visual-auditory sensory associative learning task across active learning, attention cueing learning, and passive learning modes. According to our results, the learning mode had no significant effect on learning association of congruent pairs. In addition, subjects’ performance in learning congruent samples was not correlated with their vigilance score. Nevertheless, vigilance score was significantly correlated with the learning performance of the non-congruent pairs. Moreover, in the last block of the passive learning mode, subjects significantly made more mistakes in taking non-congruent pairs as associated and consciously reported lower confidence. These results indicate that attention and activity equally enhanced visual-auditory associative learning for non-congruent pairs, while false alarm rate in the passive learning mode did not decrease after the second block. We investigated the cause of higher false alarm rate in the passive learning mode by using a computational model, composed of a reinforcement learning module and a memory-decay module. The results suggest that the higher rate of memory decay is the source of making more mistakes and reporting lower confidence in non-congruent pairs in the passive learning mode.

## Introduction

We observe the world through our multisensory system and require sensory associations across different senses play a vital role in perceiving our environment and performing high-level cognitive activities. We continually restructure already-shaped associations as we face new situations in our daily life, e.g. associating faces and voices of new individuals together, by forming novel multisensory associations. Multisensory associative learning is one of the important requirements for multisensory perception. According to one of the recent studies [1], multisensory perception is not an automatic feature-binding process and it requires an associative learning process. The learning of multisensory associations can even influence low-level sensory processing in a top-down approach[2,3]. As a result, it is expected that any improvement in multisensory associative learning would enhance our perception and cognitive abilities [4].

Many researchers have studied different aspects of human behavior, including motor learning, memory recall, and associative learning, from the perspective of active and passive involvement of subjects [5–7]. So far, the effectiveness of active learning (AL) rather than passive learning (PL) has gained the most attention in the literature, especially in uni-sensory studies [8–10]. In addition, attention is crucial in sensory processing and particularly in associative learning. Even though, the role of attention mechanism in sensory processing is not fully understood [11], there is a general consensus on its effects on sensory associative learning [12,13]. It has also been used as a treatment for tinnitus [14]. Learning when subjects receive an additional cue would enhance the learning outcome. This is referred to as Attention Cueing Learning (ACL) in this study.

Butler et al. [5,15] studied the associative learning of novel objects and sounds. They reported that AL was faster and more effective than PL. Based on the fMRI data, they showed that there are more activities in motor, somatosensory, and cerebellar regions in AL. On the other hand, it is believed that cerebellum plays important role in attention and timing [16]. Based on the aforementioned studies, it can be suggested that both AL and ACL lead to a higher activity in cerebellar region as well as an enhancement in multisensory associative learning. However, the question still remains: “which of the followings improve the multisensory associative learning more: AL or ACL?”. To the best of our knowledge, there is no unified research that compares the performance of ACL and AL in multisensory associative learning. Thereby, we have experimentally studied the effect of ACL, AL, and PL on the learning of visual-auditory sensory associations. These three modes of learning have been defined as follows: 1) Active Learning (AL) mode: a participant is responsible for pressing a key to initiate the presentation of association stimuli. 2) Attention Cueing Learning (ACL) mode: a participant’ attention is called by a cue before the presentation of association stimuli. 3) Passive Learning (PL) mode: a participant receives association stimuli without any cue or having to do any action. Comparing the performance of the participants in the AL, ACL, and PL modes would provide more insight on whether learning through attention cueing (ACL) or active learning (AL) would enhance the multisensory associative learning more.

Both the AL and ACL involve a temporal information about when the association stimulus (action-sound pair) is going to be presented. However, in AL mode, there is an additional possibility for the subject to decide when to see the association stimulus. Thus, comparing the AL and ACL would illustrate that whether deciding when to see the stimuli would enhance the associative learning. In other words, if one has access to the temporal information about an event, does having control on the occurrence time of the event enhance the associative learning of the event?

If AL and ACL enhance the multisensory associative learning equally, then they can be used interchangeably. Also, it can be speculated they use a common mechanism to enhance the multisensory associative learning. However, future brain imaging studies are required to explore this speculation. In this study, different learning modes have been computationally modeled in order to identify the cognitive processes that are potentially influenced during an associative learning task.

## Methods

### Participants

Twenty eight subjects aged between 19 and 29 years, sixteen male with mean age 23.9 and twelve female with average age 23.1, performed the experiment. Four of them were labeled as outlier because of their performance and the results of twenty four participants were considered in the analysis. All participants reported normal or corrected to normal vision, normal audition, and no neurological or psychiatric problem. The participants knew that the best three participants will receive 16 GB, 8 GB, and 4 GB flash memory, respectively. All procedures and experimental protocols are approved by the ethical committee board of University of Social Welfare and Rehabilitation Sciences, Tehran, Iran. All methods were carried out in accordance with the approved guidelines. Written informed consent was also obtained from all participants prior to data collection.

### Procedure

**Fig 1:**
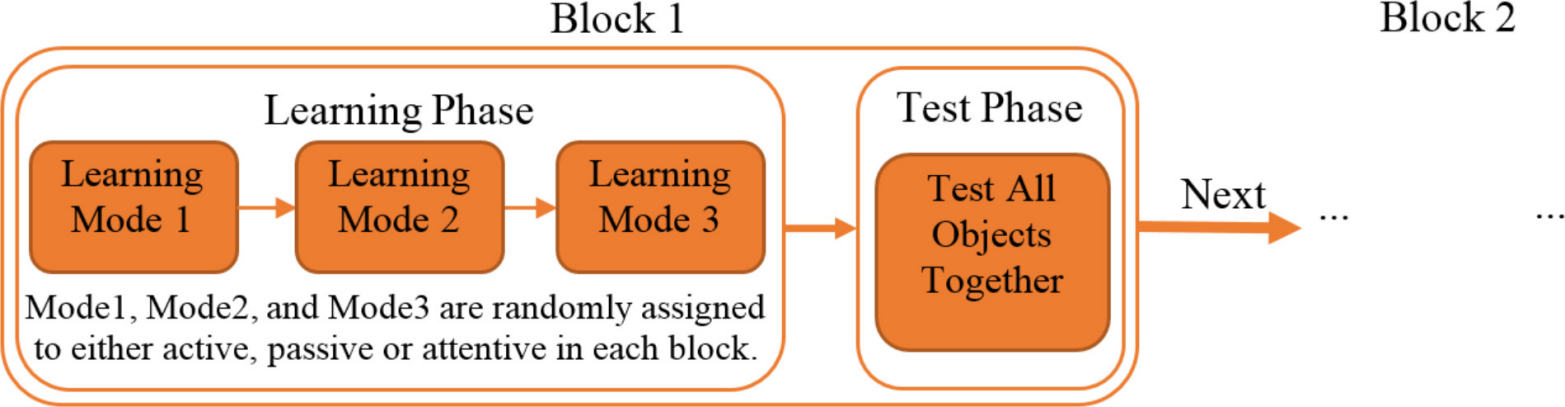
Experiment procedure. Each block consists of a learning and a test phase. In the learning phase, subjects should finish all Active Learning (AL), Attention Cueing Learning (ACL), and Passive Learning (PL) modes. The presentation order of the learning modes is determined randomly at the beginning of each block. For each subject, the object-sound pairs are assigned to the learning modes randomly at the beginning of the experiment and the assignment is kept across the blocks. After finishing the three learning modes, the subjects are evaluated in the test phase. Presentation order of the object-sound pairs is random in this phase.

The experiment was a computerized test and was designed to study the audio-visual associative learning in active, passive, and attention cueing modes. Subjects were instructed to learn associations of the 21 pairs of novel sounds and 3D objects. The experiment had four blocks, each block consisted of a learning and a test phase. In the learning phase, subjects dealt with three modes: AL, ACL, and PL. Each block included three leaning modes and ended with a test phase, where all learned associations were evaluated (See Fig 1). The order of learning modes in each block was randomly determined. In addition, three sets of seven object-sound pairs were randomly selected for each learning mode per subject. These assignments were performed at the beginning of the experiment and did not change across the blocks. This random selection was carried out to remove the effect of shape and sound biases on the subjects’ performance in the three learning modes.

Before the start of the experiment, each participant took part in a pre-test, which was exactly the same as the main experiment with just different objects and sounds. The experimenter helped participants in the pre-test in case of any question about the experiment.

Besides the designed experiment, subjects took Integrated Visual and Auditory (IVA) Continuous Performance Test (CPT) [17] to measure their vigilance capability, which is believed to play role in learning [18].

### IVA CPT

The IVA CPT experiment was implemented similar to the one mentioned by Tinius [17]. The participants were seated in the front of a monitor while they wore a headphone. They received either a green visual stimulus (1 or 2 character) or a 500ms auditory stimulus (1 or 2 sound). They were instructed to press the space key, on the keyboard, as soon as they saw or heard a “1” (target) and not to press the space key if the stimulus was a “2” (non-target). After a warm-up part, the participants received five blocks of 100 trials. In each block, during the first 50 trials, the target (“1”) was presented on 42 trials (84%) and the non-target on 8 trials (16%). In the second half of each block, the target was presented on 8 trials (16%) while the non-target was presented on 42 trials (84%). The number of the visual and auditory stimuli were balanced within each block.

### Objects and sounds

There was a pool of 21 completely novel and clearly distinct 3D objects, designed using Blender software. Objects were inscribed in a predefined cube to keep the dimensions of all objects equal. The objects subtended about 7 degrees of visual angle and were displayed in the center of a screen. The objects rotated smoothly and slowly during the presentation (6 deg/sec) to help the participants to understand the 3D shapes better. The orientation and speed of rotation were the same for all objects and across all parts of the experiment. All objects were green and the lighting condition was kept the same during the experiment.

**Fig 2:**
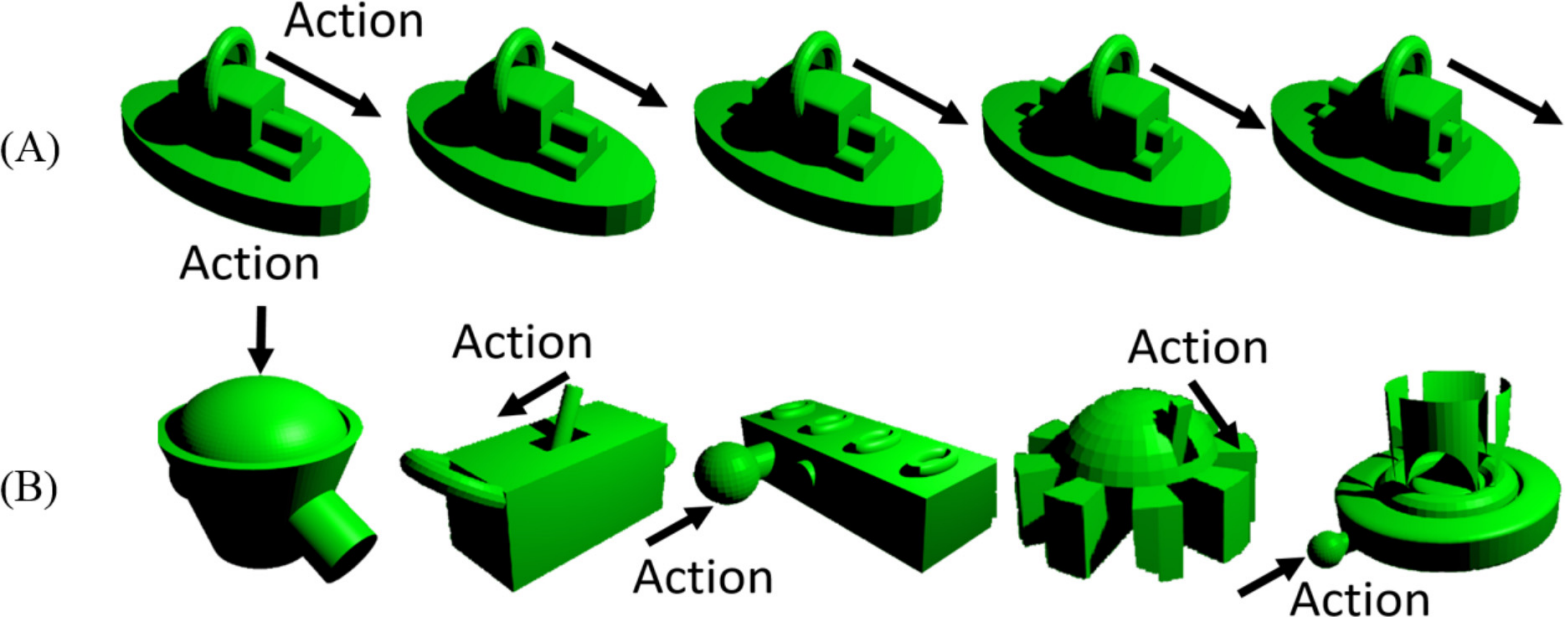
Action and objects. (A) A sample action is being performed on the object. In this example, the handle is sliding forward. (B) Five sample objects and their associated actions.

Each object has a moving part, e.g. handler, button, or slider. The displacement of the moving part creates an action, which was animated on the screen, e.g. the animation of dragging a sliding part in Fig 2.

A unique sound was associated to each object and was played while the action was being performed. The duration of all sounds was the same and was equal to the duration of the actions, which was equal to 600ms.

### Active Learning (AL) mode

**Fig 3:**
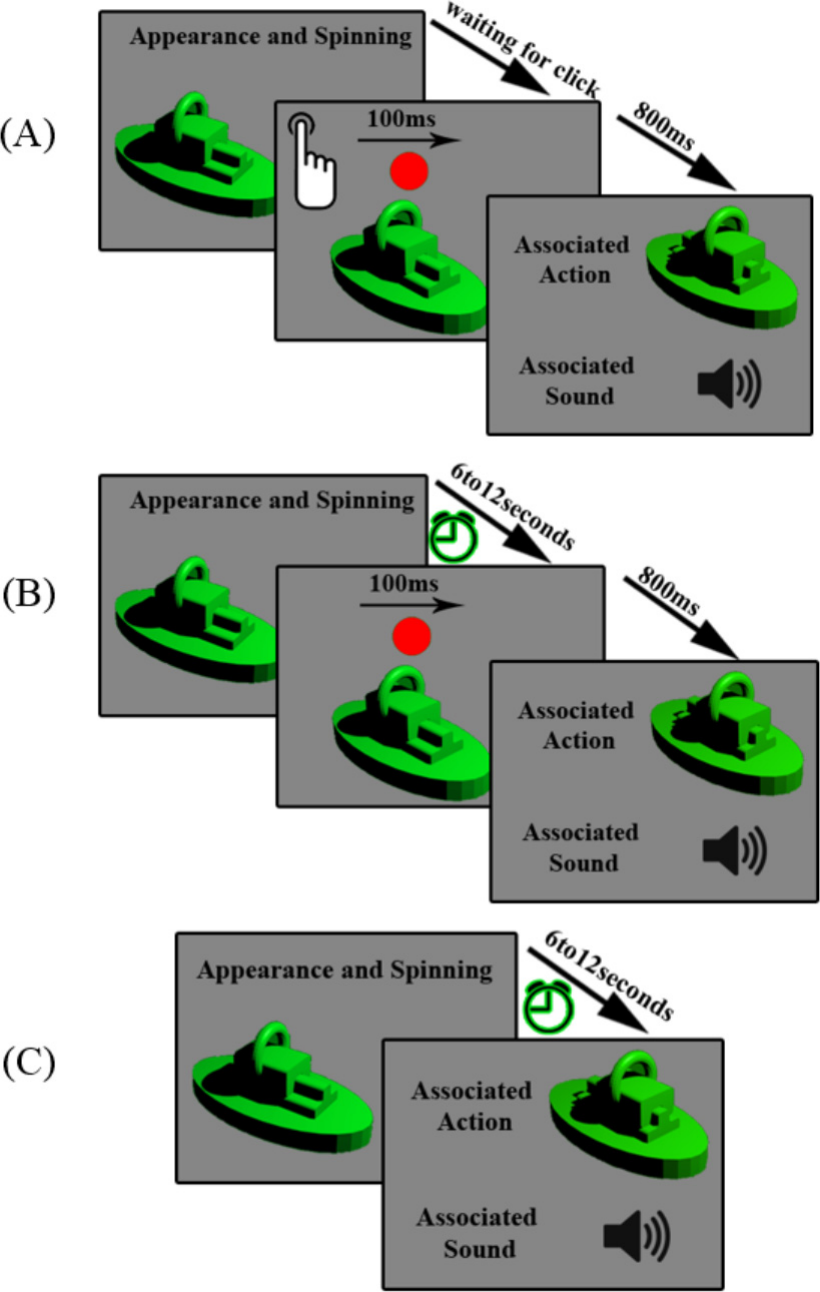
Learning modes chronology. (A) AL Mode. The object appears and begins to spin until the participant pushes the button. When he/she pushes the button, the red cue circle is shown for 100 ms and after 800 ms the associated action is performed (the moving part moves) and the associated sound is played simultaneously. (B) ACL mode. The attentive learning mode is like the active learning, but the subject has no control over the task. (C) PL mode. In this mode, the subject has no control over the task and there is also no cue before displacing the moving part and playing the sound.

In the AL mode, seven object-sound pairs were presented to each subject. The presentation order of active pairs was random. A splash screen with a short instruction was shown in the beginning of each learning mode. The presentation of the first object had started when the subject pressed the enter key. Objects were appeared in the center of the screen and instantly started to spin. There was a specific key placed in front of the subject. After pressing the key, while the object was being presented, a visual cue appeared for 100ms. 800ms after the presentation of the cue, the object’s moving part started to move while the associated sound was being played. After 800ms, the object was disappeared and the next object was appeared; see Fig 3. The visual cue was a red circle, 1.2 degrees of the visual angle size which was appeared on the screen 5.5 degrees above the center. According to the Busse’s study[19], visual cues increase attention to both visual and auditory stimuli. This procedure continued until all active pairs were presented two times. A splash screen indicated the end of the AL mode.

### Passive Learning (PL) mode

The PL mode differed from the AL mode in the subject’s control and the visualization of the cue. In this mode, the action was displayed and the sound was played at a random time between 6 to 12 seconds from the onset, without the presentation of the cue; see Fig 3. The rest of the details were similar to the AL mode.

### Attention Cueing Learning (ACL) mode

The ACL mode inherited mixed specifications of the AL and PL modes. In the ACL mode, as in the PL, subjects had no control on the timing of actions. However, subjects were cued similar to the AL mode. The cue appeared at a random time between 6 to 12 seconds from the onset. 800ms after the cue presentation, the associated action and sound were played; see Fig 3.

### Test Phase

**Fig 4:**
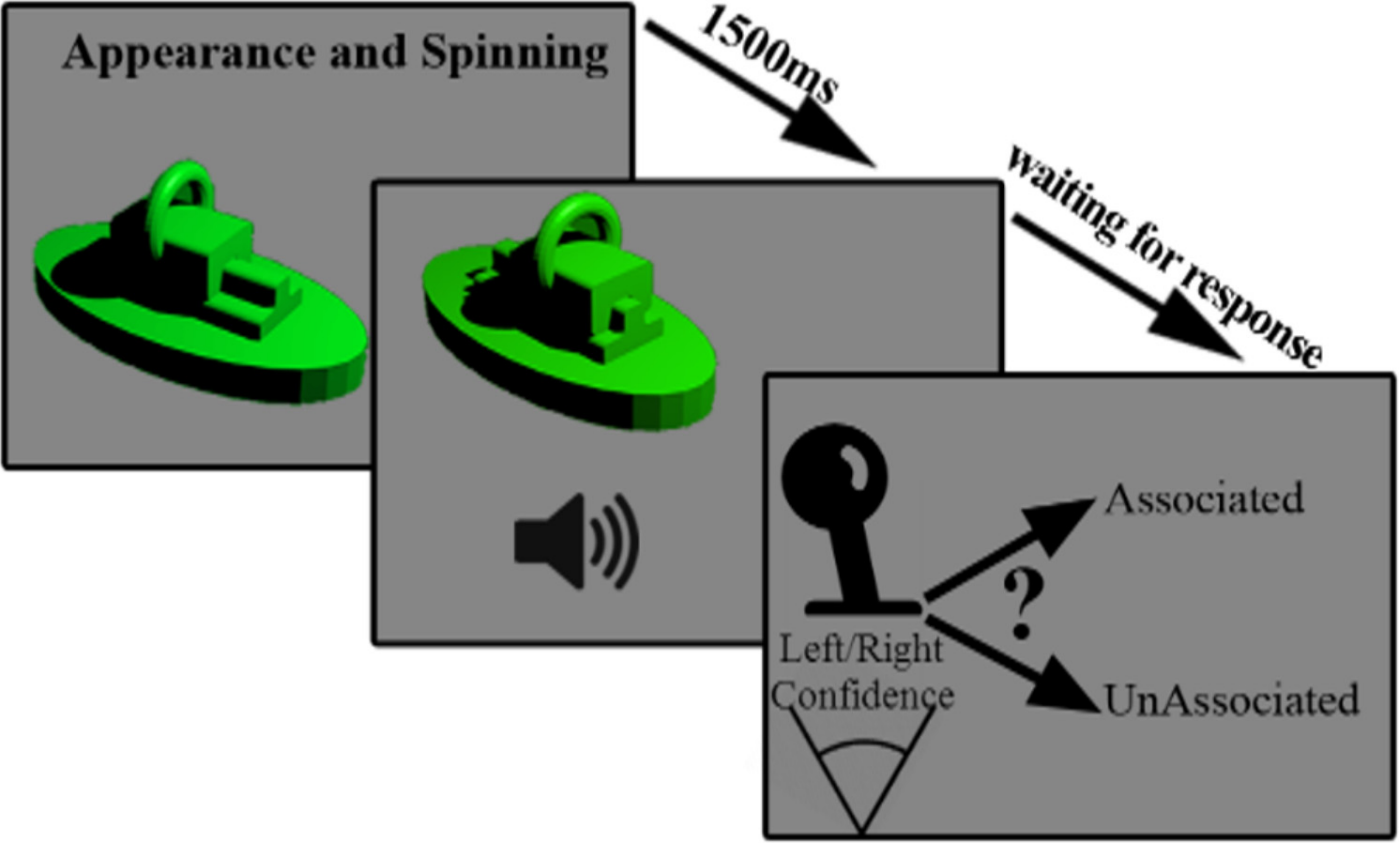
Test phase chronology. In the test phase, the object appears and starts to spin. 1500 ms later, the action is being performed while a sound is played simultaneously. The subject should determine whether the object and the sound are associated or not by choosing a corresponding direction using a joystick. The next trial begins after 1000 ms.

In the test phase, forty-two 2300ms-long movies were presented in each block. In each movie, one of the objects appeared and started to spin. After 1500ms, a sound was played while the action was being performed; see Fig 4. The sound was either associated or unassociated to the object. The subjects were asked to move a joystick to the left or right in the case of an associated or unassociated pair, respectively. The participants reported their confidence in their decision by the size of joystick movement. For example, they moved the joystick to the most right position, if they were completely confident, and moved slightly if they were not sure about their decision. The next movie was played 1000ms after the subject’s response. In the test phase, each member of the object pool was presented two times, once with the associated and once with a randomly selected unassociated sound. The presentation sequence of the associated and unassociated object-sound pairs was random.

### Experiment condition

Participants sat in a sound-attenuated room in the front of a monitor. The visual stimuli were presented through a 19” LED (40x25cm screen size) monitor with a 75Hz refresh rate. The distance between the subjects’ eyes and the monitor was 100cm. Sound stimuli were played through a stereo headphone. The openFrameworks platform was used to develop the experiment software on a windows-based PC. The experiment software was written in C++ language using Microsoft Visual Studio 2010 IDE.

### Modelling

**Fig 5:**
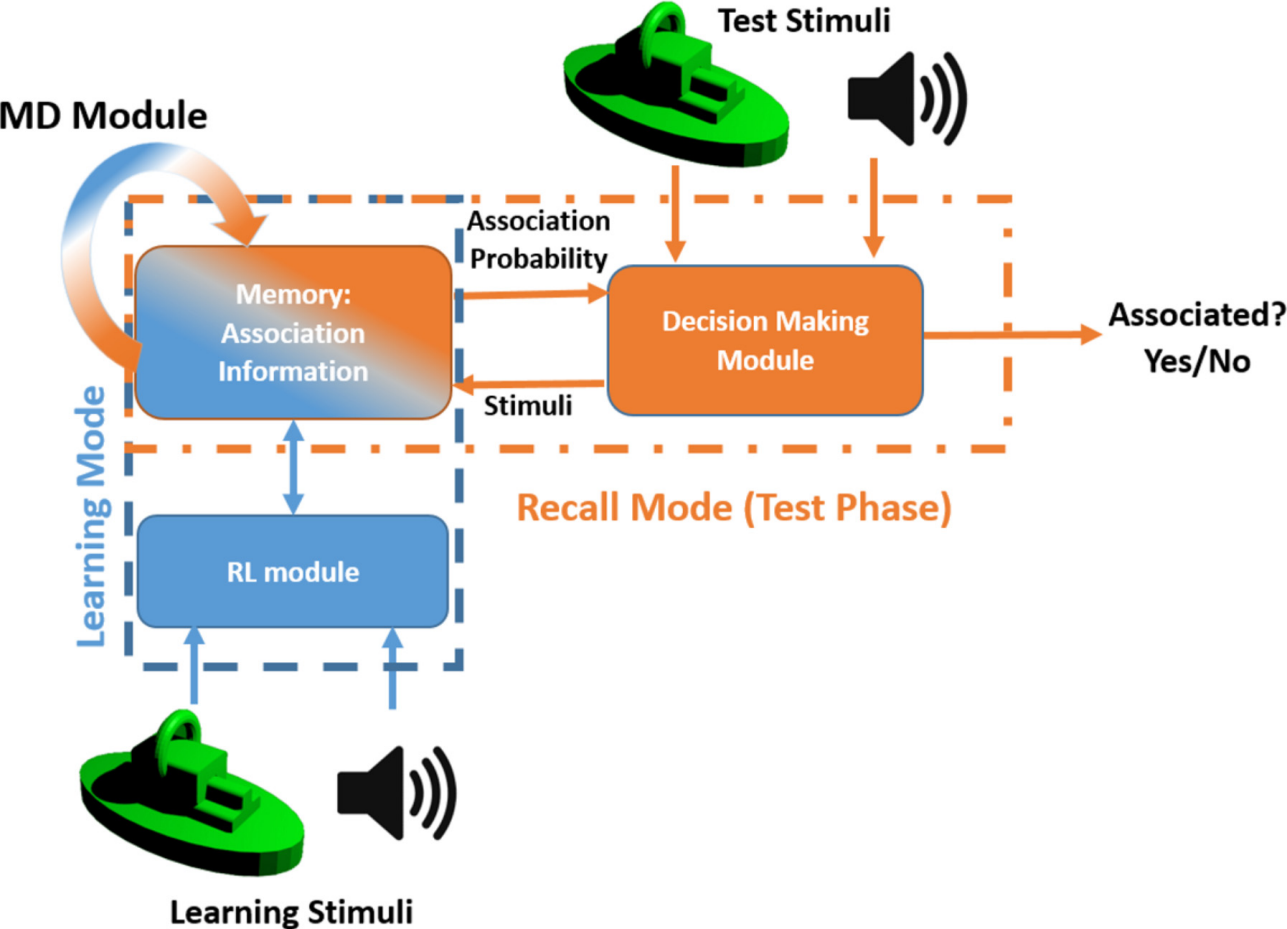
RL-MD Model. RL-MD model consists of two parts: a reinforcement learning module and a memory decay part. The model was fitted the experimental data.

The proposed associative learning task was modelled using reinforcement learning (RL) and memory decay (MD) models. Fig 5 illustrates the proposed RL-MD model in both learning and test phases. Subjective association probability of object i, O_i_, and sound j, S_j_, is denoted by P_a_(O_i_,S_j_). In the beginning of the experiment and before any observation, the association probability of all object-sound pairs are assumed to be the same and equal to $\frac{1}{{\text{N}}_{\text{sound}}}$; where N_sound_ is the number of sounds and assuming no prior information and bias in association probabilities.

In the RL module, the observation of (O_i_,S_j_) pair at time t in a learning block results in the updates of association probabilities:

$${\text{P}}_{\text{a}}{\left({\text{O}}_{\text{i}},{\text{S}}_{\text{j}}\right)}_{t}={\text{P}}_{\text{a}}{\left({\text{O}}_{\text{i}},{\text{S}}_{\text{j}}\right)}_{t-1}+\left(1-{\text{P}}_{\text{a}}{\left({\text{O}}_{\text{i}},{\text{S}}_{\text{j}}\right)}_{t-1}\right){*\alpha }_{}$$ (1)

$$\forall \;\text{k}:1\;{\text{to N}}_{\text{sound}},\;\text{k}\neq \text{j}:\;{\text{P}}_{\text{a}}{\left({\text{O}}_{\text{i}},{\text{S}}_{\text{k}}\right)}_{t}={\text{P}}_{\text{a}}{\left({\text{O}}_{\text{i}},{\text{S}}_{\text{k}}\right)}_{t-1}-\frac{1}{{\text{N}}_{\text{sound}}-1}\;\left(1-{\text{P}}_{\text{a}}{\left({\text{O}}_{\text{i}},{\text{S}}_{\text{j}}\right)}_{t-1}\right)*\alpha$$ (2)

Where P_a_(O_i_,S_j_)_*t*_ and P_a_(O_i_,S_j_)_*t*−1_ are the new and latest association probabilities, respectively; and, 0 ≤ α ≤ 1 is the learning rate parameter. We fitted α to each subject in each learning mode separately, α_*act*_, α_*att*_, and α_*pass*_ are the learning rates in the active, attention cueing, and passive modes, respectively.

The memory-decay module increases the uncertainty in decision making through decreasing/increasing the association probability of an observed/unobserved object-sound pairs in the learning phase:

$${\text{P}}_{\text{a}}\left({\text{O}}_{\text{i}},{\text{S}}_{\text{j}}\right)=\{\begin{array}{l} {\text{P}}_{\text{a}}\left({\text{O}}_{\text{i}},{\text{S}}_{\text{j}}\right)+\left(1-\text{exp}\left[-\left(1-\frac{{\text{t}}_{{\text{O}}_{\text{i}}}}{{\text{N}}_{\text{t}}}\right)\right]\right){*\beta }_{}\;if\;{\text{P}}_{\text{a}}\left({\text{O}}_{\text{i}},{\text{S}}_{\text{j}}\right)\leq 0.5\\ {\text{P}}_{\text{a}}\left({\text{O}}_{\text{i}},{\text{S}}_{\text{j}}\right)-\left(1-\text{exp}\left[-\left(1-\frac{{\text{t}}_{{\text{O}}_{\text{i}}}}{{\text{N}}_{\text{t}}}\right)\right]\right){*\beta }_{}\;if\;{\text{P}}_{\text{a}}\left({\text{O}}_{\text{i}},{\text{S}}_{\text{j}}\right)>0.5 \end{array}$$ (3)

Where ${\text{t}}_{{\text{O}}_{\text{i}}}$is the last learning trial when the object i is being observed, N_t_ is the total number of the learning trials in each block, which is equal to 42 in our experiment. β is the forgetting factor with three variants, each of them associated to one learning mode. β_*act*_, β_*att*_, β_*pass*_ are associated to the active, attention cueing, and passive modes, respectively. The forgetting factors were fitted to each subject separately.

To determine whether an object-sound pair, (O_i_,S_j_), is associated or unassociated, its subjective association probability, P_a_(O_i_,S_j_), was compared to a threshold [20]; if the probability is above or below the threshold, (O_i_,S_j_) is announced congruent or non-congruent, respectively. The threshold was fitted to each subject individually. The RL-MD model was fitted to the triples (subject, block, learning mode) using the genetic algorithm (GA). The cost function (C_k_) is the modeling error and is defined for the k^th^ participant as follows:

$${\text{C}}_{\text{k}}=\frac{\sum_{\text{i}=1}^{{\text{N}}_{\text{B}}}\sum_{\text{j}=1}^{{\text{N}}_{\text{O}}}\left|{\text{FC}}_{\text{i},\text{j}}^{}-{\text{FC}}_{\text{Mi},\text{j}}^{}\right|+\left|{\text{TC}}_{\text{i},\text{j}}^{}-{\text{TC}}_{\text{Mi},\text{j}}^{}\right|}{{\text{N}}_{\text{C}}+{\text{N}}_{\text{NC}}}$$ (4)

Where FC_Mi,j_ is the false alarm rate of the fitted model in the block i for the the j^th^ object; and, TC_Mi,j_ is the hit rate of the fitted model in the block i for the j^th^ object. FC_i,j_ and TC_i,j_ are false alarm rate and hit rate of the subject for the corresponding learning block and the object, respectively. N_B_ and N_O_ are the number of learning blocks and the number of objects, respectively. N_C_ and N_NC_ is the total number of congruent and non-congruent trials, respectively.

## Results

**Fig 6:**
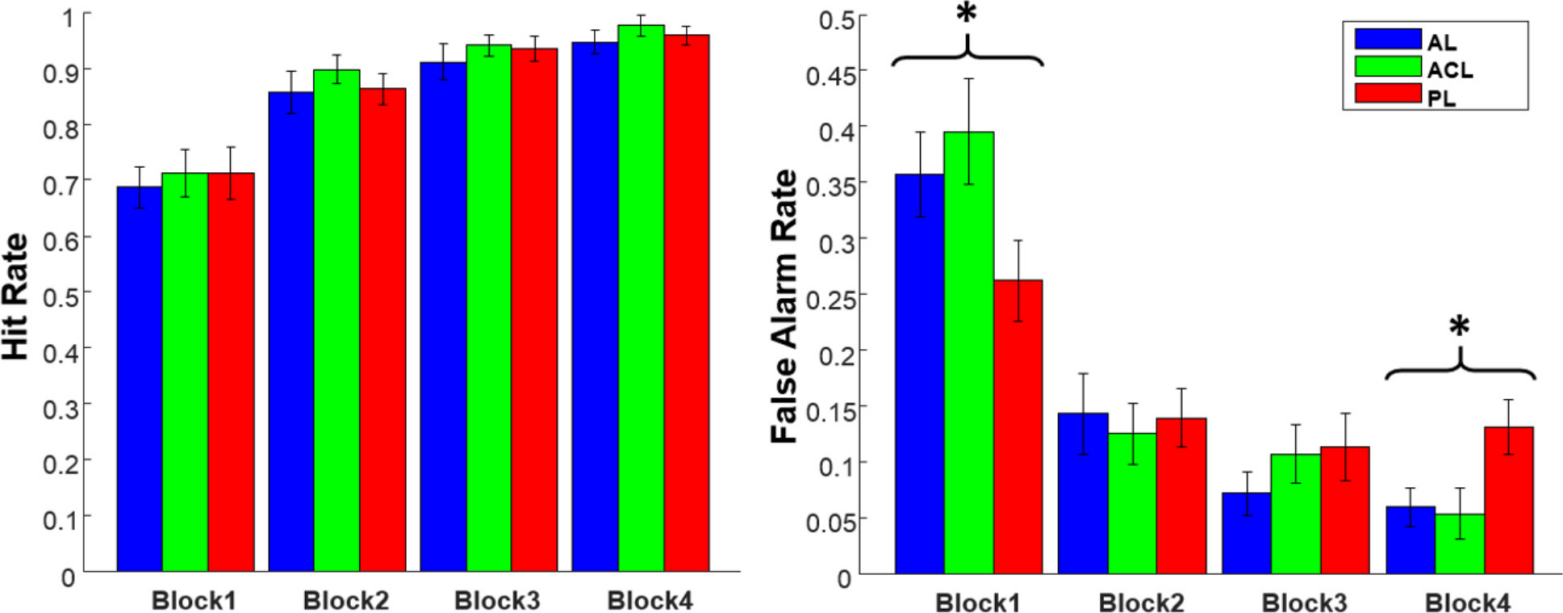
False alarm rate and hit rate. False alarm (right) and hit (left) rates in attention cueing, active and passive learning modes across four blocks. The bars show mean values and the error bars present standard error of mean. (*)ANOVA showed in the first block as well as the last block, learning modes have significantly different false alarm rate. No significant difference is observed in hit rates within the blocks.

Fig 6 shows the hit rate and the false alarm rate of the subjects in the three learning modes across four blocks. To analyze the effect of the learning mode and the learning block on the performance of the participants, hit rate and false alarm rate were analyzed using 3×4 two-way repeated ANOVA. For the hit rate (correctly announced as associated pairs), ANOVA showed a non-significant interaction effect, *F*_*(6*,*138)*_ *= 0*.*10*,*p = 0*.*99*, and a non-significant effect of the learning mode, *F*_*(2*,*46)*_ *= 1*.*12*, *p = 0*.*33*. However, a meaningful effect of learning block on hit rate was observed: *F*_*(3*,*69)*_ *= 75*.*68*, *p<0*.*0001*. For the false alarm rate (wrongly announced unassociated object-sound pairs as associated), the same as the hit rate, the effect of learning mode was non-significant *F*_*(2*,*46)*_ *= 0*.*23*, *p = 0*.*79*, and the effect of learning block was significant *F*_*(3*,*69)*_ *= 65*.*63*, *p<0*.*0001*. However, the interaction of learning block and learning mode was significant, *F*_*(6*,*138)*_ *= 4*.*01*,*p = 0*.*0009*, thus more analysis of the data is required by going through each block separately. Therefore, the effect of learning mode on false alarm rate was studied using the one-way repeated ANOVA within each learning block. Results revealed that the effect of the learning mode on false alarm rate is significant in the first block (*F*_*(2*,*46)*_ *= 3*.*96*, *p = 0*.*025*) and in the last block (*F*_*(2*,*46)*_ *= 5*.*91*, *p = 0*.*0051*). Nevertheless, for the second block (*F*_*(2*,*46)*_ *= 0*.*16*, *p = 0*.*84*) and the third block (*F*_*(2*,*46)*_ *= 1*.*17*, *p = 0*.*32*) no significant effect of the learning mode were observed. In the first block, post-hoc Tukey test showed no significant difference between AL and ACL (p = 0.62), AL and PL (p = 0.15), and ACL and PL (p = 0.055). However, in the last block, Tukey test indicated that the AL and PL were significantly different (p = 0.012), as well as ACL and PL (p = 0.011). However, no significant difference was observed between AL and ACL (p = 0.97).

By taking a glance at Fig 6, it is obvious that both the hit rate and the false alarm rate in the AL and ACL modes have the same trend in all blocks, but the subjects exhibited a different behavior in the PL mode in terms of the false alarm rate. After a rapid drop, the false alarm rate did not decrease during the last three blocks in the PL mode. Consequently, the continual decreasing trend of the false alarm rate in the AL and ACL modes, together with the unchanging false alarm rate in the PL mode during the last three blocks, lead to a significant difference in the false alarm rates across the learning modes in the last block.

Besides the features of the first order judgment, the reported confidences, as a measure of the higher order thought, were also analyzed. Similar to the false alarm rate and the hit rate, the effect of learning mode and learning block on confidence were analyzed using 3×4 two-way repeated ANOVA for congruent and non-congruent pairs separately. For congruent pairs, the interaction effect between learning mode and learning block was not significant (*F*_*(6*,*138)*_ *= 0*.*49*,*p = 0*.*81)* as well as the effect of learning mode (*F*_*(2*,*46)*_ *= 0*.*55*,*p = 0*.*57*). Nonetheless, the effect of learning block on the confidence of the congruent pairs was significant (*F*_*(3*,*69)*_ *= 101*.*2*,*p<0*.*0001*). The subjects’ confidences were also analyzed for the non-congruent pairs. The results illustrated the interaction effect between learning mode and learning block was significant (*F*_*(6*,*138)*_ *= 2*.*48*,*p = 0*.*025)*, as well as the effect of learning block (*F*_*(3*,*69)*_ *= 87*.*16*,*p<0*.*0001*), but the effect of the learning mode was not significant (*F*_*(2*,*46)*_ *= 0*.*36*,*p = 0*.*70*). The significant interaction indicating that more analysis within each block was required. Therefore, the confidences within each block were compared across the learning modes, using the one-way repeated ANOVA as follows: *F*_*(2*,*46)*_ *= 2*.*14*, *p = 0*.*12* for the first block, *F*_*(2*,*46)*_ *= 0*.*08*, *p = 0*.*91* for the second block, *F*_*(2*,*46)*_ *= 1*.*01*, *p = 0*.*37* for the third block, and *F*_*(2*,*46)*_ *= 4*.*93*, *p = 0*.*01* for the last block.

**Fig 7:**
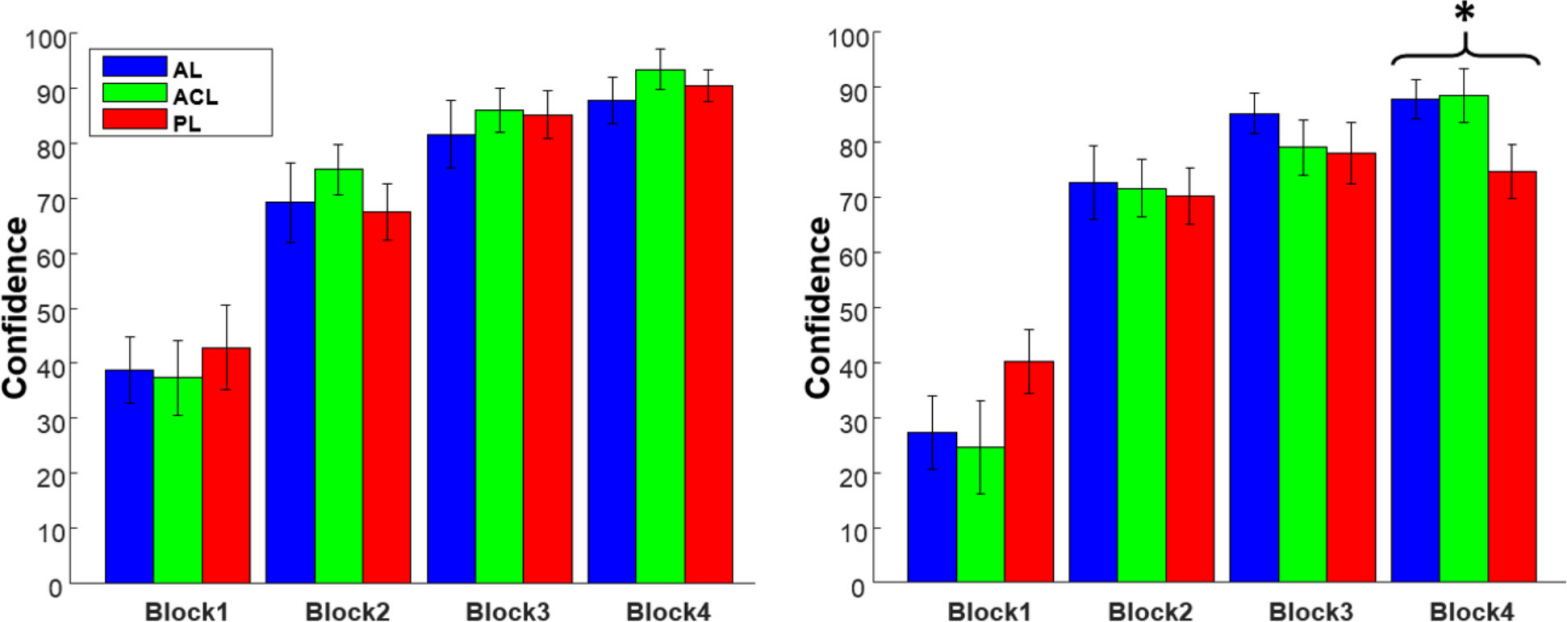
Confidence reporting. The subjects’ average confidence in their decisions for congruent (left) and non-congruent (right) pairs. The confidence values have an increasing trend except for non-congruent passive pairs in the last block. In this block (*), the subjects are significantly less confident for non-congruent passive pairs.

Fig 7 illustrates that confidences for non-congruent PL pairs did not change in the last three blocks, while the confidences for non-congruent AL and ACL pairs increased gradually. The higher false alarm rate and lower confidence in the last block of the PL mode suggests that participants performed worse while they were aware of their own performance. A close to perfect performance in the last block of the AL and ACL modes, increasing trend in the hit rate, and decreasing trend in the false alarm rate (except the false alarm rate in the PL mode) show the sufficiency of the subjects’ learning capacity for this task. Besides, since all of three learning modes were performed in all four blocks in a random order, fatigue cannot be accounted as the source of observed difference in the last block.

Similar to judgments and confidences, reaction times were analyzed for congruent and non-congruent groups separately. Two-way repeated ANOVA indicated non-significant interaction between learning mode and learning block in both congruent (*F*_*(6*,*138)*_ *= 1*.*35*,*p = 0*.*23*) and non-congruent (*F*_*(6*,*138)*_ *= 1*.*06*,*p = 0*.*38*) pairs. The effect of the learning block on the reaction time was significant for congruent pairs (*F*_*(3*,*69)*_ *= 22*.*92*,*p<0*.*0001*) as well as for non-congruent pairs (*F*_*(3*,*69)*_ *= 31*.*89*,*p<0*.*0001*), is suggesting the learning caused a decrease in the reaction time. However, the effect of learning mode on reaction time was not significant, neither for congruent pairs (*F*_*(2*,*46)*_ *= 1*.*97*, *p = 0*.*15*) nor for non-congruent pairs (*F*_*(2*,*46)*_ *= 1*.*97*, *p = 0*.*15*).

The correlation between the IVA CPT error (vigilance error) and the judgment’s features, i.e. false alarm rate and hit rate, was analyzed using linear mixed-effects model with a varying intercept and varying slope for each learning mode. The fixed independent variables of the model were the false alarm rate and the hit rate; and, the dependent variable assumed to be IVA CPT error. For false alarm rate, the intercept was 0.02 (*F*_*(1*,*70)*_ *= 0*.*001*, *p = 0*.*96*) and the slope was 2.20 (*F*_*(1*,*70)*_ *= 11*.*81*, *p = 0*.*0009*). However, the intercept was 5.54 (*F*_*(1*,*70)*_ *= 5*.*87*, *p = 0*.*017*) and the slope was negative -1.17 (*F*_*(1*,*70)*_ *= 3*.*23*, *p = 0*.*076*) for the hit rate. The random effects of the false alarm rate and the hit rate were not significant; thus, no significant difference was observed across the learning modes. The significant positive correlation (slope) between the false alarm rate and the IVA CPT error and the lack of the significant correlation between the hit rare and IVA CPT error indicate that vigilant people improve their associative learning by reducing the false alarm rate.

### Reinforcement Learning-Memory Decay Model

**Fig 8:**
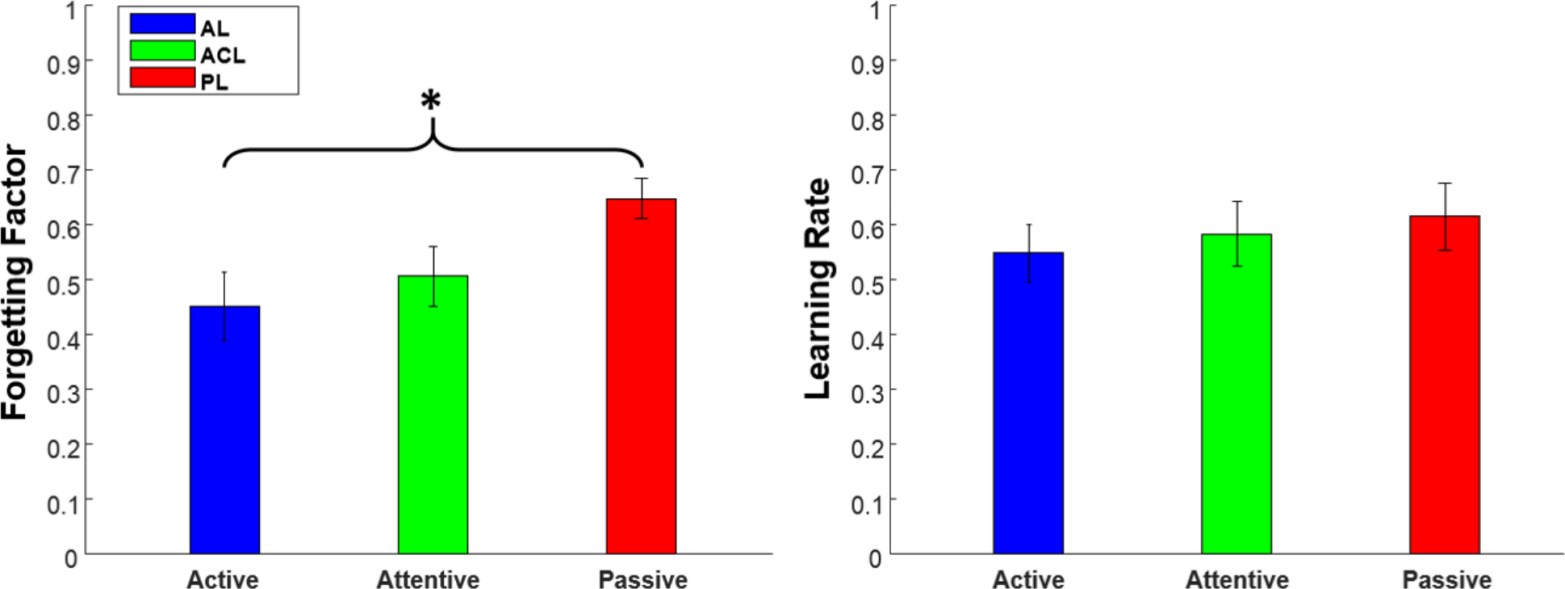
Modelling parameters. Forgetting factor (left) and learning rate (right) in active, attention cueing, and passive learning modes. The bars show mean values and the error bars present standard error of mean. (*) The forgetting factor in the passive mode is significantly higher.

We modelled our associative learning task by a mixture of a reinforcement learning (RL) module [21] and a memory-decay (MD) module (see Fig 5). The role of the RL is to assign experienced-based values to associated pairs in the learning phase, and to keep the values in memory for decision making in the test phase. In the decision making part, a subjective and value-based selection probability is assigned to each object-sound pair. The memory-decay module models forgetfulness through increasing the uncertainty in decision making in a time-exponential [22] manner. In other words, RL and MD work in opposite directions; RL increases the certainty in decision making by gathering experience in the learning phase while MD results in higher uncertainty by time. Therefore, our model helps to know whether the differences in AL, ACL, and PL modes are caused by learning parameters or by memory decay. The absolute modelling errors over all participants were 0.1518±0.08, 0.1628±0.10, and 0.1583±0.11 for AL, ACL, and PL modes, respectively. Fig 8 shows the fitted learning rates and the forgetting factors across learning modes. One-way repeated ANOVA illustrated that the difference in forgetting factor across AL, ACL, and PL modes was significant (*F*_*(2*,*46)*_ *= 3*.*53*, *p = 0*.*037*), while the learning rate was not significantly different across the learning modes (*F*_*(2*,*46)*_ *= 0*.*38*, *p = 0*.*68*). This suggests that the source of the diversity in performance across the learning modes is MD, not RL. However, additional brain imaging experiments are required to observe the brain’s activity during the learning in AL, ACL and PL modes.

## Discussion

Associative learning has a prominent role in our daily life, in both real and virtual environments, and particularly in multisensory integration [1]. Multisensory associative learning is also known as the first and primary step of multisensory perception. It can even influence low-level sensory processing in a top-down manner [2]^,^[3]. Therefore, finding means to increase the quality of associative learning is the goal of some researchers.

A few papers [15] show that active learning, in comparison to passive learning, results in higher performance in multisensory associative learning. It is also reported that attention cueing results in higher performance in associative learning [3]. These results point at inferiority of passive learning in terms of learning performance. Nevertheless, the performance of multisensory associative learning in the two modes, attention cueing learning and active learning, has not been studied. In addition, the source of the difference in the performance across the three modes, AL, ACL and PL, is not still clear enough.

In this research, we investigated the performance of subjects in a visual-auditory associative learning experiment across AL, ACL, and PL in four consecutive blocks. The results show that there is no significant difference between the subjects in terms of recalling the congruent object-sound pairs, i.e. they have similar hit rates for AL, ACL and PL modes. In addition, there is no significant difference in the subjects’ confidence in their decisions for congruent pairs across the learning modes. Nevertheless, the false alarm rate in the PL mode does not change in the last three blocks, while it has a decreasing trend in the AL and ACL modes. Therefore, the subjects have a significantly higher false alarm rate in the last block of the PL mode, i.e. they wrongly announced non-congruent pairs as associated more often in the last block of the PL mode. The reported confidences are in line with the false alarm rates that suggests the participants were aware of their own performance. The confidences for non-congruent pairs in the PL mode only increase significantly in the second block and it has no changes in the later blocks. However, the confidences for non-congruent pairs in the AL and ACL modes increase moderately in the last three blocks. Therefore, the subjects were comparatively less confident about their decisions over non-associated pairs in the PL mode in the last block. These results suggest that recalling the congruent pairs in visual-auditory associative learning is independent of learning mode, while it is not the case for the non-congruent pairs. In addition, the IVA CPT error has a correlation with the false alarm rate. However, it does not have any correlation with the hit rate. This implies the absense of vigilance role in the learning of congruent pairs.

Our results show that there is no significant difference between the AL and ACL modes in terms of hit rate, false alarm rate, and response time. These similarities in various aspects of the subjects’ response suggest that the AL and ACL modes might exploit the same mechanism to increase associative learning performance. However, further brain imaging study is needed to test this speculation.

It is widely assumed that, learning and memory are the key components in multisensory associative learning tasks. Accepting this assumption, we hypothesize that AL, ACL, and PL influences subjects’ performance through these two components. In our task, the subjects observed congruent visual-auditory pairs during the learning phases. Close to perfect hit rates in the last block and similar increasing trend across all learning modes suggest that the modes do not affect the learning of the congruent pairs. In addition, the subjects had no chance to observe non-congruent pairs in the learning phase. Therefore, learning cannot be accounted for the difference in false alarm rates. Now, the question is whether the memory can be accounted for higher false alarm rate in the last block of the PL mode. To investigate this question, we used a computational model composed of a reinforcement learning (RL) module, to learn congruent pairs, and a memory decay (MD) module, to keep the association probabilities of all visual-auditory pairs. The MD module models forgetfulness as an exponential increase of uncertainty in associations. In other words, RL and MD work in the opposite directions. RL increases certainty in the association of congruent pairs by gaining experience in the learning phase, while MD results in higher uncertainty as time passes. The modeling results indicate that there is no significant difference in the learning rates across the modes. Nevertheless, the PL mode has a higher forgetting factor in comparison to ACL and AL. This higher memory decay justifies the lower confidence in the last block of non-congruent pairs in the PL mode.

Although we know that the competition between memorizing items leads to forgetting [23], recent studies in both real [24] and virtual environments [25] indicate an improvement in memory decay when subjects are actively performing a task. Schomaker et al. [25] showed that activity in a novel virtual environment can improve recall even on unrelated word learning task. The results also suggest that novelty can improve attention in virtual environments more than in the real one [25]. Trewartha et al. [24] revealed that the recall in the real world improves when subjects actively explore objects. Voss et al. [7] illustrates that volitional exploration during learning can enhance memory performance in comparison with passive learning. They showed that volitional control can optimize interaction among hippocampus and other areas. These results are in line with our modeling outcome, better performance in the AL and ACL modes is due to the better memory recall. Our model suggests that the effect of the learning mode on incongruent pairs can be explained by its influence on the memory decay rather than the learning process. Thus, improving the memory is a beneficial way to reduce the false alarm rate, probably without impacting on the hit rate. In other words, the AL and ACL equally reduce the false alarm rate through improving memory. It also shows if one has access to the temporal information about the occurrence time of an association stimulus, the memory recall improves in terms of the false alarm rate. Thus, accessing the temporal information about the occurrence of an event is sufficient to improve the memory, independent of the learning mode. We speculate that active learning may modulate attention to access the temporal information and that results in lower memory decay compare to passive learning in multisensory associative learning tasks.
